# Gecko-Inspired Biocidal Organic Nanocrystals Initiated from a Pencil-Drawn Graphite Template

**DOI:** 10.1038/s41598-018-29994-3

**Published:** 2018-08-02

**Authors:** David L. Gonzalez Arellano, Kristopher W. Kolewe, Victor K. Champagne, Irene S. Kurtz, Edmund K. Burnett, Julia A. Zakashansky, Feyza Dundar Arisoy, Alejandro L. Briseno, Jessica D. Schiffman

**Affiliations:** 1 Department of Polymer Science & Engineering, University of Massachusetts Amherst, Amherst, Massachusetts 01003-9303 USA; 2 Department of Chemical Engineering, University of Massachusetts Amherst, Amherst, Massachusetts 01003-9303 USA; 3 Department of Chemistry, The Pennsylvania State University, University Park, PA 16802 USA

## Abstract

The biocidal properties of gecko skin and cicada wings have inspired the synthesis of synthetic surfaces decorated with high aspect ratio nanostructures that inactivate microorganisms. Here, we investigate the bactericidal activity of oriented zinc phthalocyanine (ZnPc) nanopillars grown using a simple pencil-drawn graphite templating technique. By varying the evaporation time, nanopillars initiated from graphite that was scribbled using a pencil onto silicon substrates were optimized to yield a high inactivation of the Gram-negative bacteria, *Escherichia coli*. We next adapted the procedure so that analogous nanopillars could be grown from pencil-drawn graphite scribbled onto stainless steel, flexible polyimide foil, and glass substrates. Time-dependent bacterial cytotoxicity studies indicate that the oriented nanopillars grown on all four substrates inactivated up to 97% of the *E. coli* quickly, in 15 min or less. These results suggest that organic nanostructures, which can be easily grown on a broad range of substrates hold potential as a new class of biocidal surfaces that kill microbes quickly and potentially, without spreading antibiotic-resistance genes.

## Introduction

Globalization, an overreliance on commercial antibiotics, and a decline in antibiotic discovery have resulted in widespread bacterial antibiotic resistance. Antibiotic-resistant bacteria, a global healthcare crisis, annually infect more than two million people in the United States leading to 23,000 associated deaths^1–3^. Thus, the development of new technologies that can combat bacterial infections without facilitating the spread of resistant bacteria is one of the preeminent challenges of the twenty-first century. Because contact-biocidal surfaces kill microorganisms using a direct chemical or physical interaction, which does not release antimicrobial agents, they hold potential as a promising alternative to commercial antimicrobials^4–6^. Non-leachable antimicrobials, such as cationic charged groups (i.e., quaternary amines, cationic alkoxysilanes, *N*-alkylated polyethyleneimine), have been extensively employed in contact-biocidal surfaces due to their long-lasting antimicrobial activity and ability to disrupt bacterial cell membranes through chemical interactions^7–11^. While non-leachable, charge-based antimicrobials are currently effective, given sufficient time bacteria could potentially gain resistance to these antimicrobials, necessitating the development of new and alternative bactericidal materials^12–14^.

The physical bactericidal mechanism observed on the cicada wings and gecko skin provides inspiration for the development of new materials that could inactivate bacteria. Cicada wings and their synthetic mimics (i.e., black silicon) are surfaces decorated with a regular array of short pillars (50–70 nm in diameter) that are spaced ~200 nm apart; they can kill Gram-negative bacteria, Gram-positive bacteria, and endospores independent of their chemical composition^15–17^. Interestingly, the skin of the box-patterned gecko (*Lucasium* sp.) is decorated with dome shaped scales arranged in a hexagonal patterning, which kill Gram-negative bacteria^18^. The gecko’s scales are comprised of spinules (hairs) that are much larger than the cicadas’ pillars, (several hundred nanometers to several microns in length) with sub-micron spacing and a small radius of curvature (10 to 20 nm)^18^. Because the gecko’s spinules have a wider spacing and are significantly longer than the structures on cicada wings, previous reports have noted that if the same inactivation mechanism employed on gecko skin was valid on cicada wings, then fewer structural contacts would be needed to stretch the cell’s membrane thus impairing cell function and causing death^18^. Notably, the exact “contact killing” mechanism, as well as the optimal geometry and density of nanostructures for killing bacteria is not fully understood^19^. To-date, no experimental evidence has demonstrated that bacteria can physically remodel their cellular exterior to gain “resistance” towards these physical killing mechanisms^15,20–24^.

In this work, we use pencil-drawn graphite to initiate high-aspect-ratio single crystalline nanopillars from the organic semiconductor material zinc phthalocyanine (ZnPc) to mimic the spinules found on geckos. A new pencil-drawn graphite templating technique was used on silicon (Si) substrates to determine the optimal evaporation time for producing surfaces covered with bactericidal nanopillars. Scanning electron microscopy (SEM) was used to characterize the dimensions of the nanopillars, including their length, base diameter, and top diameter. After the nanopillar growth conditions that maximized microbial inactivation were optimized on Si wafers, we produced analogous nanopillars on glass, flexible polymer, and stainless steel substrates. By growing nanopillars initiated from pencil-drawn graphite instead of from the conventional CVD produced graphene templating layer, our findings present a straightforward, facile, substrate independent, and inexpensive^25^ method of fabricating bioinspired surfaces that reduce bacterial contamination in lieu of commercial antimicrobial agents.

## Results

### Characteristics of organic semiconductor nanostructures

**Figure 1:**
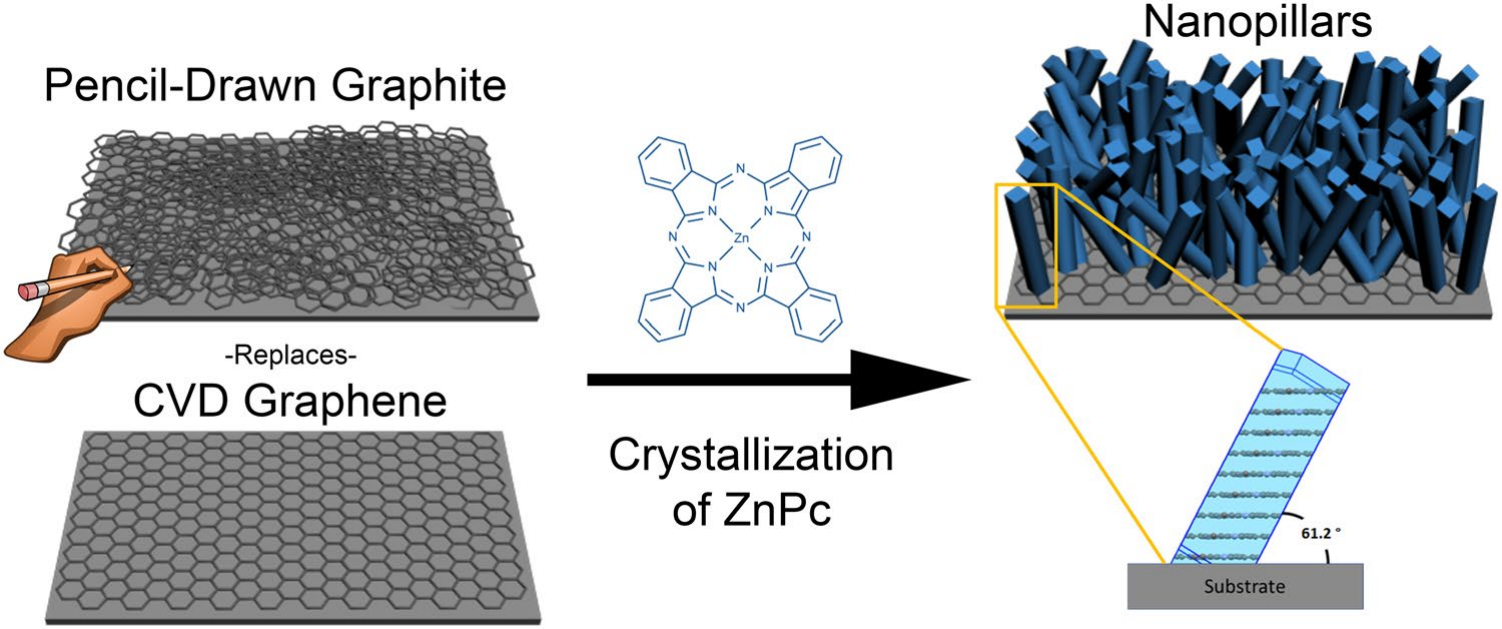
Schematic of zinc phthalocyanine (ZnPc) nanopillars initiated from pencil-drawn graphite and from a conventional graphene obtained via chemical vapor deposition (CVD). After the templating layer is prepared, physical vapor transport (PVT) is used to evaporate the organic material. The result is an array of oriented ZnPc nanopillar crystals with random azimuthal orientation. Single crystals of ZnPc stack in a semi-vertical orientation with the substrate at a 61° angle. Substrates used in this manuscript include Si wafer, stainless steel, flexible polyimide foil, and glass.

The schematic in Fig. 1 describes the pathway for fabricating oriented nanopillars on pencil-drawn graphite versus the conventional CVD graphene surfaces using a physical vapor transport (PVT) crystallization method. Here, we replaced the graphene^26,27^ commonly used by scribbling on silicon (Si) wafers using a pencil (graphite bar 8B) purchased from the University of Massachusetts Amherst art store. This pencil-drawn graphite served as the templating interlayer (RMS = 1.94 nm, Supplementary Fig. S1) before depositing the organic semiconductor material zinc phthalocyanine (ZnPc)^28,29^. For nanopillar growth, a graphite-coated Si wafer was placed inside a PVT apparatus under vacuum (10^−2^ mbar) and the ZnPc was heated at its sublimation temperature (420 °C) to maintain a deposition rate of approximately 3 Å/s. By holding the substrate at 350 °C, the incoming molecules adsorbed to the graphite surface through π-π interactions, growing in a layer-by-layer fashion forming an ultra-thin two-dimensional “wetting layer.” Beyond a critical thickness of about 5–10 nm, the growth continues through a strain-induced process forming adsorbate “islands” which grow into nanopillars. Further details of the nanopillar growth mechanisms that occur when CVD graphene is used as the template layer can be found in our recent publication^27^.

**Figure 2:**
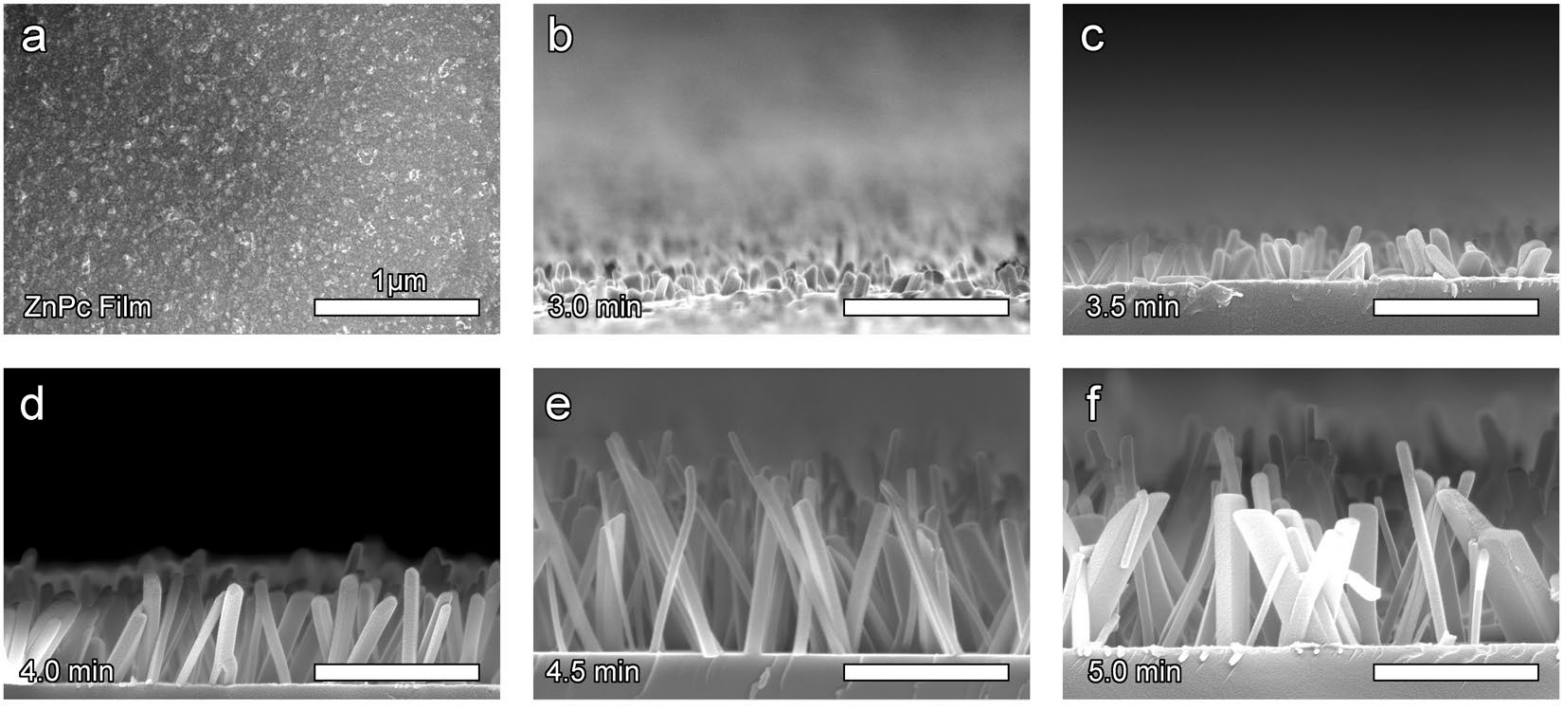
Micrographs of nanopillars initiated from pencil-drawn graphite as a function of evaporation time. (**a)** ZnPc film deposited on Si wafer (no nanopillars). (**b–f**) Cross-sectional micrographs of ZnPc nanopillars initiated from pencil-drawn graphite grown as a function of evaporation time, from 3.0 min to 5.0 min. All scale bars are 1 µm.

During their growth, dimensions of the organic crystals are controlled through the selection of the organic source material, the underlying template layer, and the deposition parameters (i.e., rate, temperature, and time). In this work, the deposition parameters, rate, and temperature, were held constant to investigate the effect of growth time on ZnPc nanopillar morphology and the corresponding microbial inactivation. The scanning electron micrograph (SEM) in Fig. 2a is a control ZnPc film (20 nm thin, no nanopillars) fabricated using thermal evaporation on a Si wafer at room temperature. The ZnPc films, the chemistry control for the antimicrobial work, consists of polycrystalline grains approximately 40 nm in diameter with a surface roughness of approximately 6 nm^30^.

**Figure 3:**
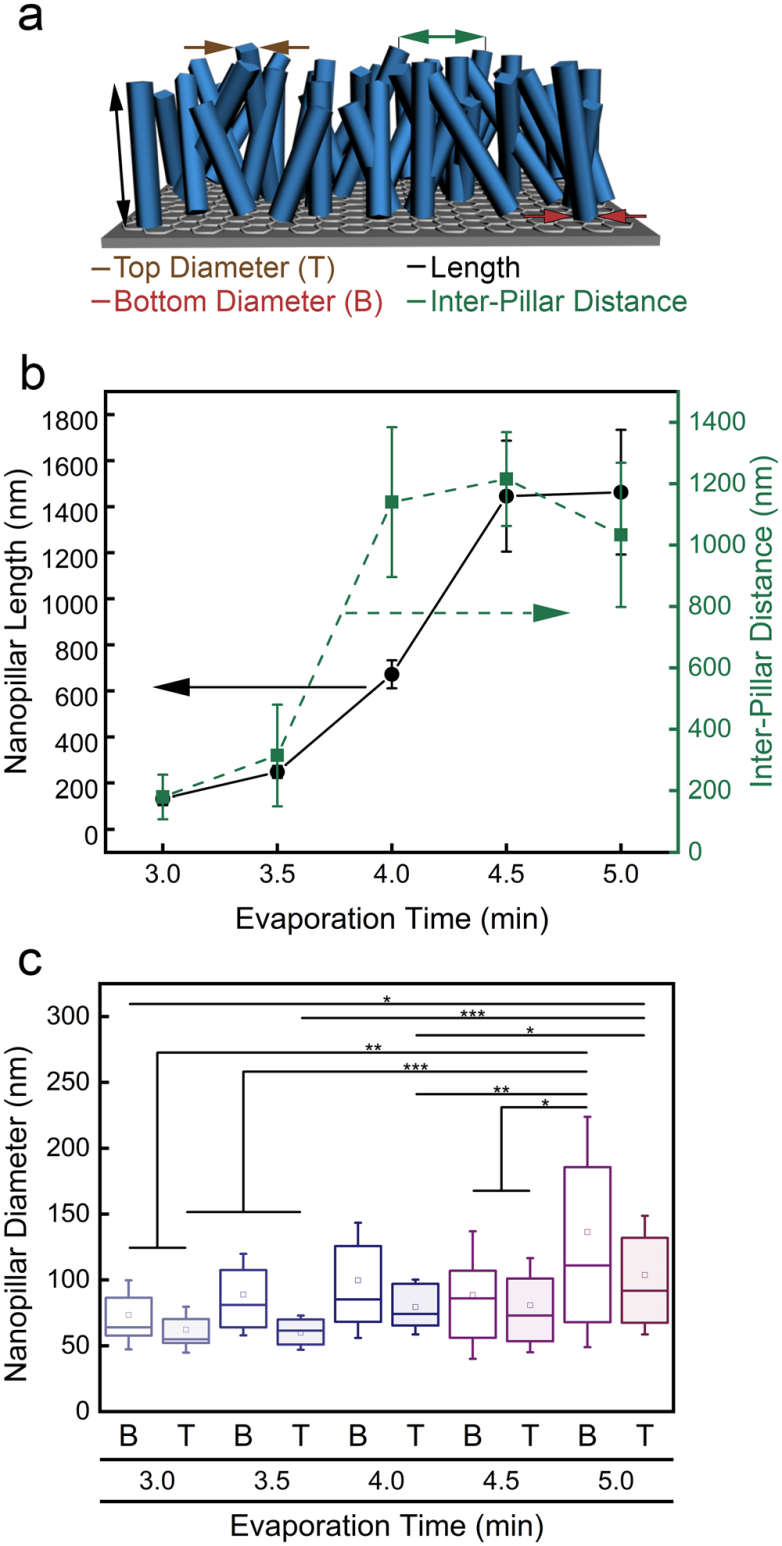
Dimensions of nanopillars initiated from pencil-drawn graphite as a function of evaporation time. (**a**) Cartoon of the nanopillars and dimensions measured, including top diameter, bottom diameter, length, and inter-pillar distance. (**b)** Nanopillar length (left, black circles) and inter-pillar distance (right, green squares) as a function of evaporation time. Standard deviation is provided. (**c**) Nanopillar bottom (B) and top (T) diameter as a function of evaporation time. The box outline is the standard error, the square inside the box is the mean data point, and the line inside the box is the median data location. One asterisk (*) indicates that values are significantly different at 0.05 level, two asterisks (**) indicates that values are significantly different at 0.01 level, and three asterisks (***) indicates that the values are significantly different at 0.001 level.

The micrographs in Fig. 2b–f show the growth of the ZnPc nanopillars as a function of evaporation time with 30 sec intervals. The nanopillar length increases by an order of magnitude, from 132 ± 27 nm to 1446 ± 241 nm at 3.0 min and 4.5 min, respectively, Table 1 and Fig. 3. Notably, there is a decreased growth rate for the last 0.5 min (4.5 min versus 5.0 min). Consistent with previous reports^31^, the nanopillar diameters are 10 to 30% wider at the base than at the top of the nanopillars. For example, after 5.0 min of evaporation, nanopillars have a base and top diameter of 136 ± 85 nm and 104 ± 44 nm, respectively. Statistically speaking, most of the nanopillars have the same bottom and top diameter, with the nanopillars grown for 5.0 min having the widest variance.

**Table 1:** Dimensions of nanopillars initiated from pencil-drawn graphite as a function of evaporation time.

Evaporation time (min)	Length (nm)	Bottom diameter (nm)	Top diameter (nm)	Inter-pillar distance (nm)
3.0	132 ± 27	74 ± 26	62 ± 17	180 ± 72
3.5	248 ± 157	89 ± 30	60 ± 13	315 ± 26
4.0	672 ± 80	100 ± 43	79 ± 20	1140 ± 61
4.5	1446 ± 241	89 ± 47	81 ± 35	1215 ± 153
5.0	1463 ± 271	136 ± 85	104 ± 44	1033 ± 235

Previous reports note that the geometry and variations in the shape of the nanostructures can influence the bacterial-killing efficiency^32–34^. The inter-pillar spacing changes with evaporation time and the length of the nanopillars. If the inter-pillar distance is too large compared to the bulk height of the nanopillars, then it is possible that the bacteria will accumulate on the flat surface between the pillars, drastically decreasing the bactericidal effect^35^. Conversely, reports have shown that decreasing the distance between nanopillars could lead to an increase in bactericidal efficiency^36,37^. In Fig. 3b, the inter-pillar distance is displayed alongside nanopillar length as a function of evaporation time. ZnPc nanopillars grow with azimuthal freedom following the molecular packing dictated at the template interface. As a result, there is a statistical variation of inter-pillar distances reported at various deposition times. However, given the density of the nanopillars coupled with their inter-pillar distance versus *E. coli*’s size (~1.0 µm × 0.5 µm), it is unlikely that bacteria could transport through the bulk of the nanopillar coating.

### Biocidal activity of nanopillars initiated from pencil-drawn graphite

**Figure 4:**
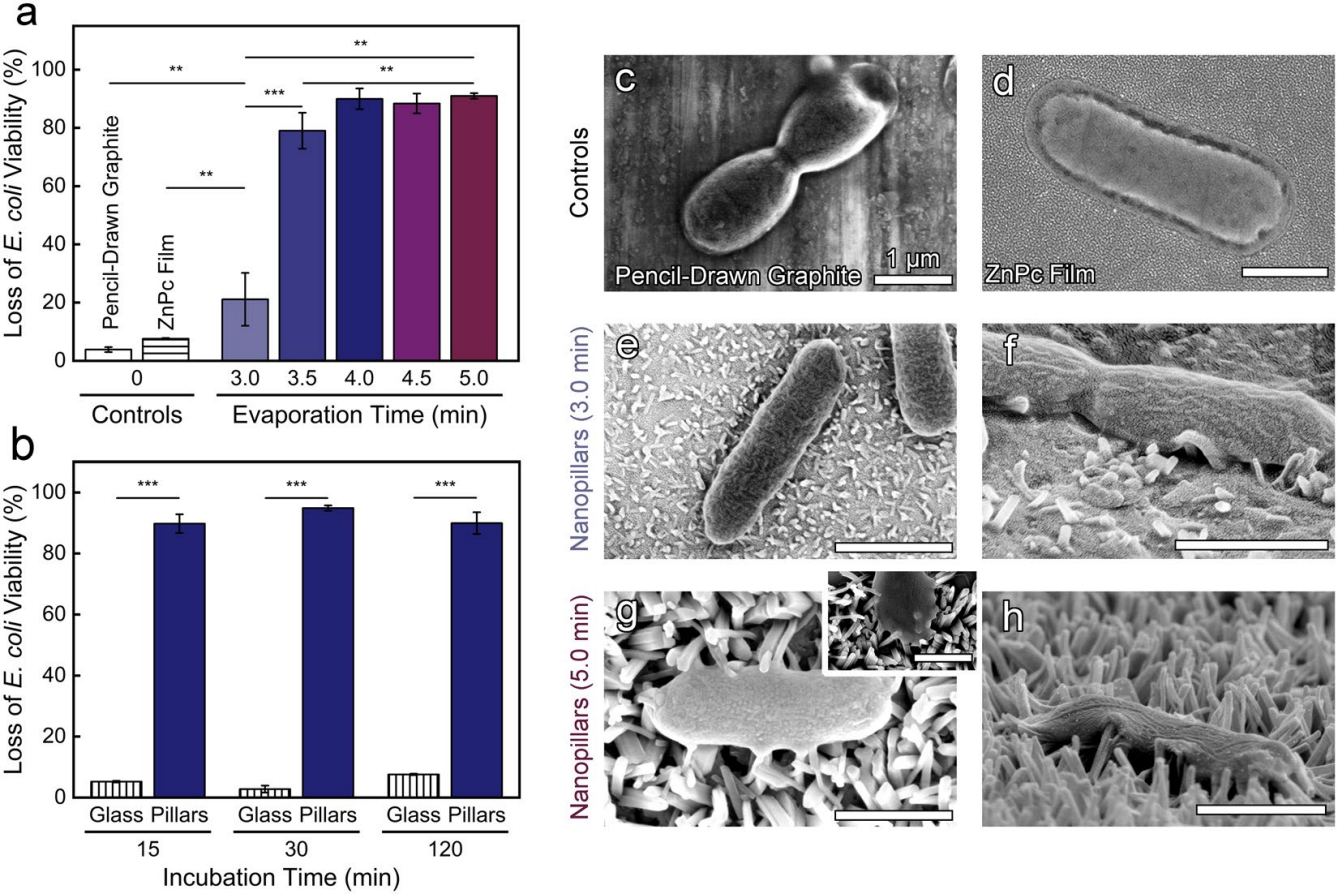
Nanopillars initiated from pencil-drawn graphite (for an evaporation time of 4.0 min) kill microorganisms within 15 min. (**a**) Viability of *E. coli* after a 2 hr incubation on nanopillars initiated from pencil-drawn graphite as a function of evaporation time. Pencil-drawn graphite (no nanopillars) and ZnPc film (no nanopillars) controls are provided. (**b)** Viability of *E. coli* incubated on nanopillars initiated from pencil-drawn graphite as a function of incubation time. All nanopillars were grown for an evaporation time of 4.0 min. (**a**,**b)** Standard error is provided. Two asterisks (**) indicates that values are significantly different at 0.01 level and three asterisks (***) indicates that the values are significantly different at 0.001 level. SEM micrographs of *E. coli* incubated on control samples (no nanopillars), (**c)** pencil-drawn graphite, and (**d)** ZnPc film. Top-down and side-profile SEM micrographs of *E. coli* incubated on (**e**,**f**) short and (**g**,**h**) long nanopillars. The inset micrograph (**g**) highlights contact points between the nanopillars and an *E. coli*. All scale bars are 1 µm.

In Fig. 4 and Supplementary Fig. S2, the bactericidal efficacy of the nanopillars initiated from pencil-drawn graphite were compared to control materials, including ZnPc films and a film of pencil-drawn graphite (no nanopillars). As expected, the control flat films demonstrate a very low, baseline loss of *E. coli* viability of 4.0 ± 0.9% and 8.0 ± 0.2%, for the pencil-drawn graphite and ZnPc films, respectively. The SEM micrographs (Fig. 4c,d) visually corroborate that *E. coli* maintained their characteristic rod-like morphology on the chemistry controls indicating that the chemistry of the materials did not have inherent bactericidal properties.

The presence of nanopillar topography caused a statistical increase in the loss in *E. coli* viability. Nanopillars fabricated using the shortest evaporation time of 3.0 min resulted in a 21 ± 9% killing efficiency, while a slightly longer evaporation time of 3.5 min exhibited a statistically greater killing efficiency of 79 ± 6%. Variations in growth time and the corresponding changes in length and inter-pillar spacing have a significant impact on the killing efficiency during the 3.0 and 3.5 min evaporation time, whereas the peak killing was reached after growing the nanopillars for a 4.0 min evaporation time. We observed that both nanopillar growth and bactericidal activity plateau after 4.0 min of crystal growth. The nanopillars grown for an evaporation time of 4.0, 4.5, and 5.0 min achieved a statistically equivalent killing efficiency of ~90%.

Notably, while most biophysical models suggests how bacteria might be inactivated by the nanotopography on cicada wings^37,38^, our materials more closely mimic the skin of geckos. The inset in Fig. 4g is provided to show contact points between the nanopillars and the *E. coli* that suggest signs of cell deformation. Because of their extremely narrow diameter, single walled carbon nanotubes have been reported to pierce the membranes of bacteria^24^, but the diameter of our ZnPc nanopillars are much larger (~100 nm). Thus, it is highly unlikely that our nanopillars are piercing the microbes. As noted by Watson *et al*.^17^, different gecko species have spinules with various heights and a wide range of spacings, from ~200 nm to over 700 nm, which is very similar to the inter-pillar spacing that we report (~200 nm to 10000 nm). The back scales of the *L. steindachneri* gecko were typically 100–190 µm in diameter and while our pillars are narrower, we suggest that our organic nanocrystals mimic the key features of gecko skin that are needed to provide an antimicrobial functionality.

To investigate the kinetics of microbial inactivation, we systematically varied the time that *E. coli* were incubated on nanopillars grown using the most effective evaporation time (4.0 min), Fig. 4b and Supplemental Fig. S3. After 15 min of contact, the shortest time interval that could be tested using the fluorescence-based toxicity assay, a 90 ± 3% loss in *E. coli* viability was achieved. This is statistically equivalent to the loss of *E. coli* viability after 120 min, 90 ± 4%, suggesting that *E. coli* die quickly when in contact with the nanostructured surface. The short time required for killing is similar to reports on the *Psaltoda claripennis* (cicada wings) where the killing was evident after 20 min^15^.

### Characteristics and biocidal activity of nanopillars initiated from pencil-drawn graphite on stainless steel, flexible polyimide foil, and glass substrates

**Figure 5:**
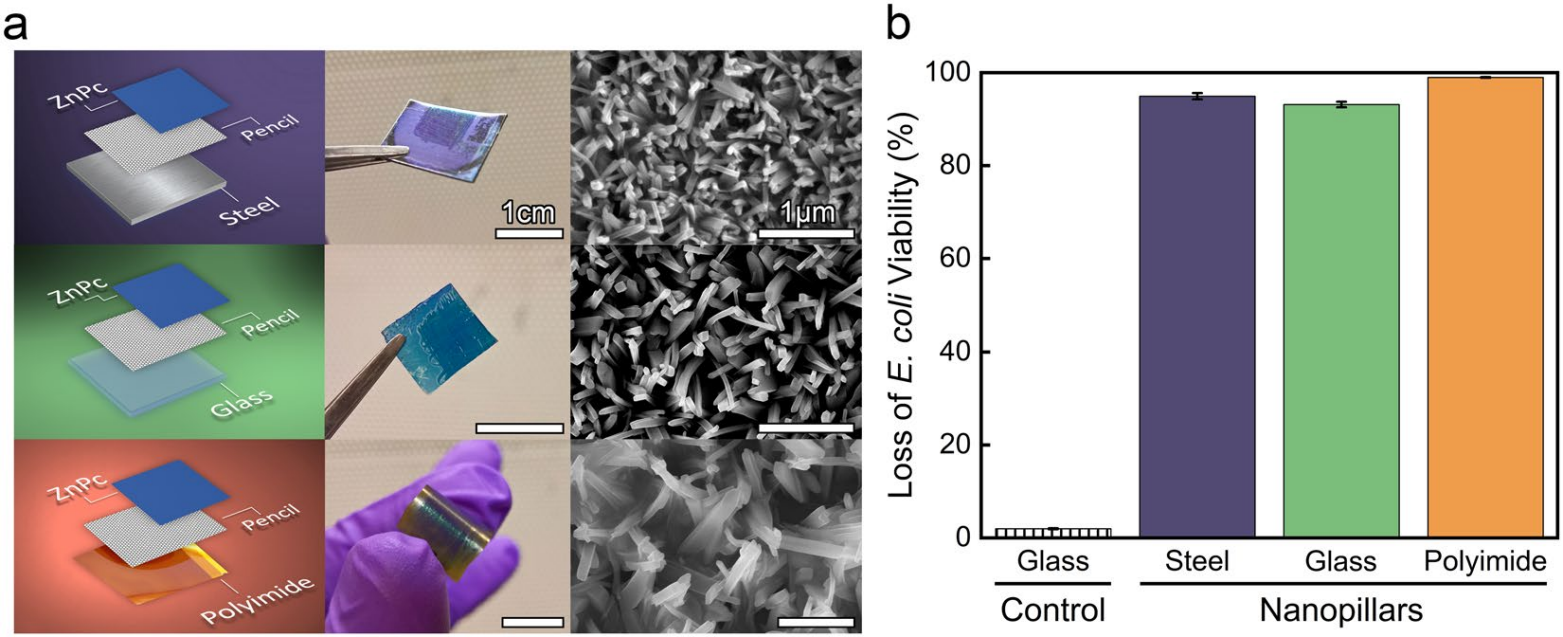
Nanopillars initiated from pencil-drawn graphite scribbled on stainless steel, polyimide, and glass substrates kill microbes within 15 min. (**a**) Pencil-drawn graphite was used as the templating layer on stainless steel, polyimide, and glass substrates. Dark blue areas on the digital images display where pencil-drawn graphite was applied, 1 cm scale bars provided. SEM micrographs display the nanopillars, 1 µm scale bars provided. (**b**) Viability of *E. coli* after a 15 min incubation on nanopillars initiated from pencil-drawn graphite. All nanopillars were grown using a 4.0 min evaporation time. Standard error is provided.

To develop a versatile, economic platform for bactericidal nanopillars, we investigated if our method of nanopillar growth initiated from pencil-drawn graphite was applicable to a variety of substrates. Indeed, nanopillars grew via a pencil-drawn graphite that was scribbled on stainless steel, flexible polyimide foil, and glass, Fig. 5a. For successful nanopillar growth, the adhesion of graphite flakes to the underlying substrate needed to be improved by first roughening the substrate by wet sanding (using sand paper with a grain size of 3 μm) prior to hand-drawing using pencil. Using the optimal evaporation time of 4.0 min, all surfaces displayed oriented crystallization on top of the graphitic regions, indicated qualitatively by the darker shade of blue on each substrate in Fig. 5a.

The efficacy and kinetics of the bactericidal nature of the pencil-drawn graphite nanopillars was investigated by varying the incubation time of *E. coli* on nanopillars grown on stainless steel, glass, and polyimide substrates. The biocidal characteristics of nanopillars grown on the various substrates reflect a similar behavior to those grown on Si substrates. For both the 120 min and the quick, 15 min incubation times, there was at least a 97 ± 3% loss in *E. coli* viability achieved regardless of the underlying substrate (Fig. 5b and Supplemental Figs S4 and S5). In contrast, only one microorganism was inactivated when the *E. coli* were incubated on control glass samples for 120 min. These examples show the versatility of pencil-drawn graphite and its adaptability to a plethora of substrates, thus opening the door for growing biocidal nanopillars on a diverse range of substrates from metals to flexible polymer films.

## Conclusion

This paper is the first report that demonstrates antibacterial surfaces featuring high aspect-ratio organic single-crystal nanopillars. We show that the geometry of ZnPc nanopillars greatly influences bactericidal performance. Antibacterial tests with *E. coli* on our nanoengineered surfaces proved lethal to the bacteria with a 97% killing efficiency without the use of external forces or antibiotics. Current work on oriented organic semiconductors is typically conducted using expensive and time-consuming 2D materials, such as CVD graphene. In this report, however, pencil-drawn graphite is demonstrated as a viable and inexpensive method for growing oriented nanostructures on a variety of substrates. This work establishes a new class of biocidal surfaces that inactivate microorganisms that does not rely on release antimicrobials.

## Materials and Methods

### Materials

All compounds were used as received. Zinc phthalocyanine dye content 97%, phosphate buffered saline (PBS, 1 × sterile biograde), propidium iodide (PI), Luria−Bertani broth (LB), M9 minimal salts (M9 media), D-(+)-glucose, calcium chloride (anhydrous), and ampicillin (BioReagent grade) were purchased from Sigma-Aldrich (St. Louis, MO). Copper foil (0.025 mm thick) and Puratronic® (99.999%) were acquired from Alfa Aesar. Deionized (DI) water was obtained from a Barnstead Nanopure Infinity water purification system (Thermo Fisher Scientific, Waltham, MA). Silicon (Si) [100] (boron doped, resistivity <0.005Ω • cm) was purchased from Addison Engineering, Inc (San Jose, CA). Pencil (graphite bar 8B) was purchased from the University of Massachusetts Amherst art store.

### Nanopillar fabrication

Throughout the manuscript, nanopillars were initiated from pencil-drawn graphite. The substrate (Si wafers, glass, polyimide, or steel) were roughened by wet sanding using sand paper (grain size of 3 μm), which enabled graphite flakes to attach to the substrate after pencil was hand-scribbled on its surface. The growth of the nanopillars was performed using physical vapor transport (PVT) in a vacuum tube with the source material placed at the base of the apparatus. The substrate was held 5 cm above the source material with the graphitic face in the direction of the molecular beam. The system was evacuated to a base pressure of 10^−2^ mbar and heated to the source material sublimation temperature. The apparatus consists of a sublimation zone and a deposition zone; the sublimation zone was heated to 420 °C and the temperature decreases gradually towards the deposition zone to 350 °C. Nanopillar height was varied by changing the evaporation time from 3.0 to 5.0 min. Graphene reference samples were grown on copper foil via chemical vapor deposition (CVD) and transferred to Si wafers. Zinc phthalocyanine films (control samples) were fabricated by thermal evaporation under vacuum at a base pressure of 5 × 10^−6^ mbar. The Si wafer was held at room temperature (21 °C).

### Nanopillar characterization

Micrographs of nanopillars were acquired using an FEI Magellan 400 XHR scanning electron microscope (SEM, Hillsboro, OR). A Cressington 108 HR sputter coater (Cressigton Scientific Instruments, Watford, England) was used to coat samples with 5 nm of gold. The nanopillar length, inter-pillar distance, base diameter and top diameter distribution were determined by measuring 10 nanopillars from 3 micrographs using *ImageJ* 1.45 software (National Institutes of Health, Bethesda, MD).

### Antibacterial activity characterization

The bactericidal efficiency of substrates was evaluated using a modified adhesion viability assay^39^. *Escherichia coli* K12 MG1655 (*E. coli*) (DSMZ, Leibniz-Institut, Germany) containing a GFP plasmid were cultured overnight in LB media, then washed with and resuspended in M9 minimal media. Control and nanopillar substrates were placed at the base of 6-well plates (Fisher Scientific) to which 5 mL of M9 media containing 100 µg/mL of carbenicillin was added to select for GFP expressing *E. coli* (1 × 10^8^ cells/mL). Samples were incubated in the dark at 37 °C for a predetermined incubation period then removed and rinsed lightly with M9 media to remove non-adhered cells. Internal controls (glass coverslips) were run in parallel. PI stain (15 min) identified the dead cells, while GFP expressing *E. coli* were considered viable. The loss of *E. coli* viability was visualized using a Zeiss Microscope Axio Imager A2M (Thornwood, NY), quantified using *ImageJ 1.48* software, and the percentage of dead cells (or loss of viability) was determined from the ratio of the number of cells stained with PI divided by the total number of cells.

### Statistics

Significant differences between samples were determined with an unpaired Student *t*-test. Significance is denoted in the graphs using asterisks and defined in the figure captions.

### Data availability

The data that support the findings of this study are available from the corresponding author upon reasonable request.

## Electronic supplementary material

Supplemental Information
