# Supplementary material for: Gecko-Inspired Biocidal Organic Nanocrystals Initiated from a Pencil-Drawn Graphite Template
Source: Sci Rep. 2018 Aug 2;8:11618. doi: 10.1038/s41598-018-29994-3 (PMC6072760; doi:10.1038/s41598-018-29994-3)
Supplement: Supplementary file 1 — Supplemental Information [file 41598_2018_29994_MOESM1_ESM.pdf]

Gecko-Inspired Biocidal Organic Nanocrystals Initiated from a Pencil-Drawn Graphite Template

David L. Gonzalez Arellano[1†], Kristopher W. Kolewe[2†], Victor K. Champagne III[1†], Irene S. Kurtz[2], Edmund K. Burnett[1], Julia A. Zakashansky[1], Feyza Dundar Arisoy[1], Alejandro L. Briseno*[1,3], and Jessica D. Schiffman*[2]

† These authors contributed equally.

1. Department of Polymer Science & Engineering, University of Massachusetts Amherst, Amherst, Massachusetts 01003-9303, USA.

2. Department of Chemical Engineering, University of Massachusetts Amherst, Amherst, Massachusetts 01003-9303, USA.

3. Department of Chemistry, The Pennsylvania State University, University Park, PA 16802, USA.

*Corresponding authors: alb818@psu.edu and schiffman@ecs.umass.edu

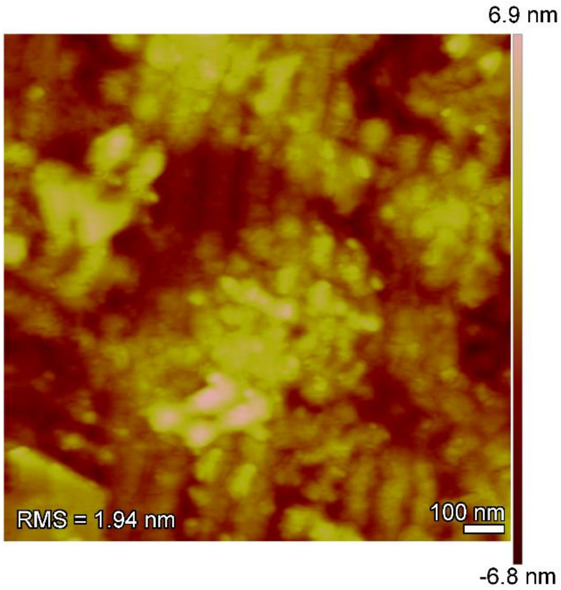

Figure S1. Atomic force microscopy image of pencil-drawn graphite on a silicon (Si) wafer, root mean square roughness is 1.94 nm. A z-scale is provided.

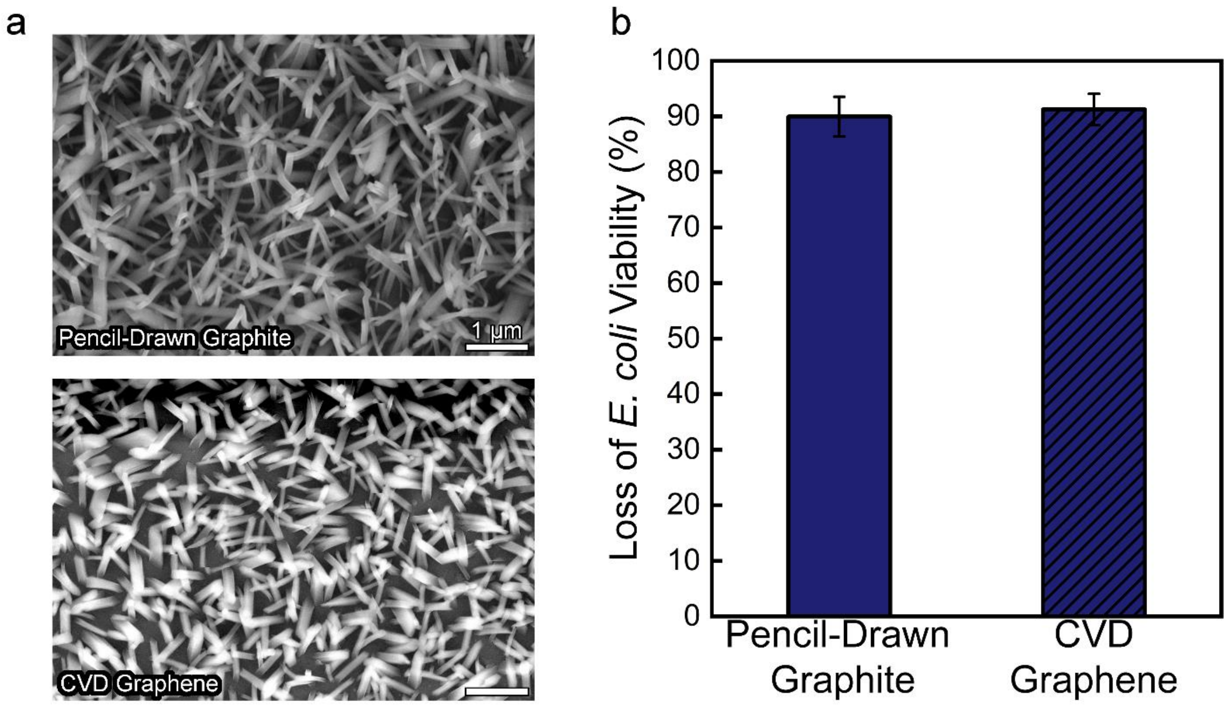

Figure S2. a Representative scanning electron micrographs of zinc phthalocyanine (ZnPc) nanopillars initiated from pencil-drawn graphite and initiated from conventional graphene-based template obtained via chemical vapor deposition (CVD). Provided scale bars are 1 µm. **b** Viability of *E. coli* after a 2 hr incubation on nanopillars initiated from pencil-drawn graphite and on a conventional graphene-based template. All nanopillars were grown for an evaporation time of 4.0 min. Error bars denote standard error.

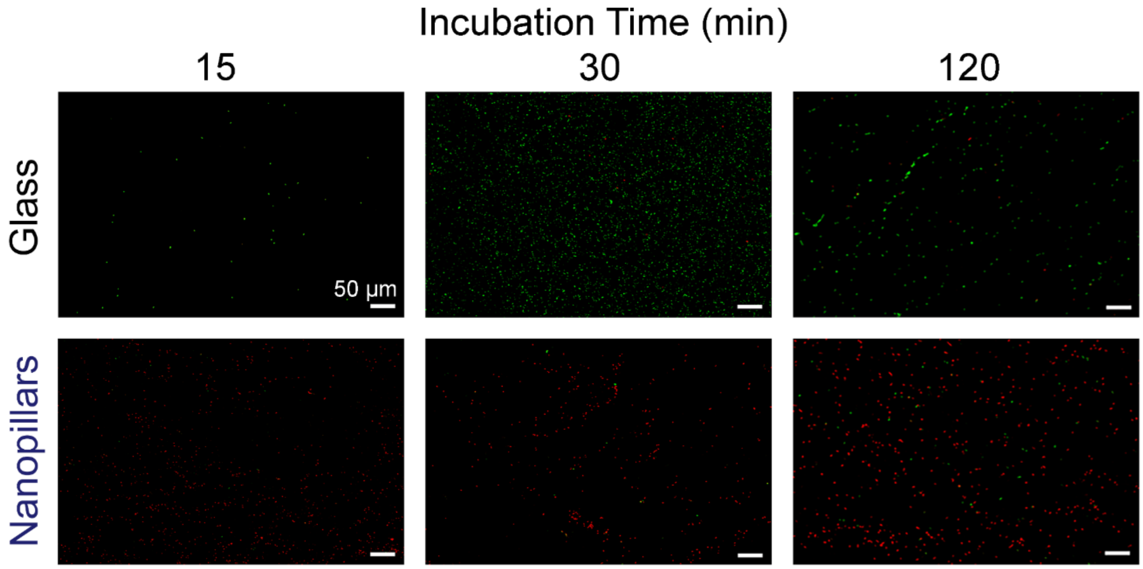

Figure S3. Representative micrographs of *E. coli* incubated on nanopillars initiated from pencil-drawn graphite as a function of time. All nanopillars were grown for an evaporation time of 4.0 min. Provided scale bars are 50 μm.

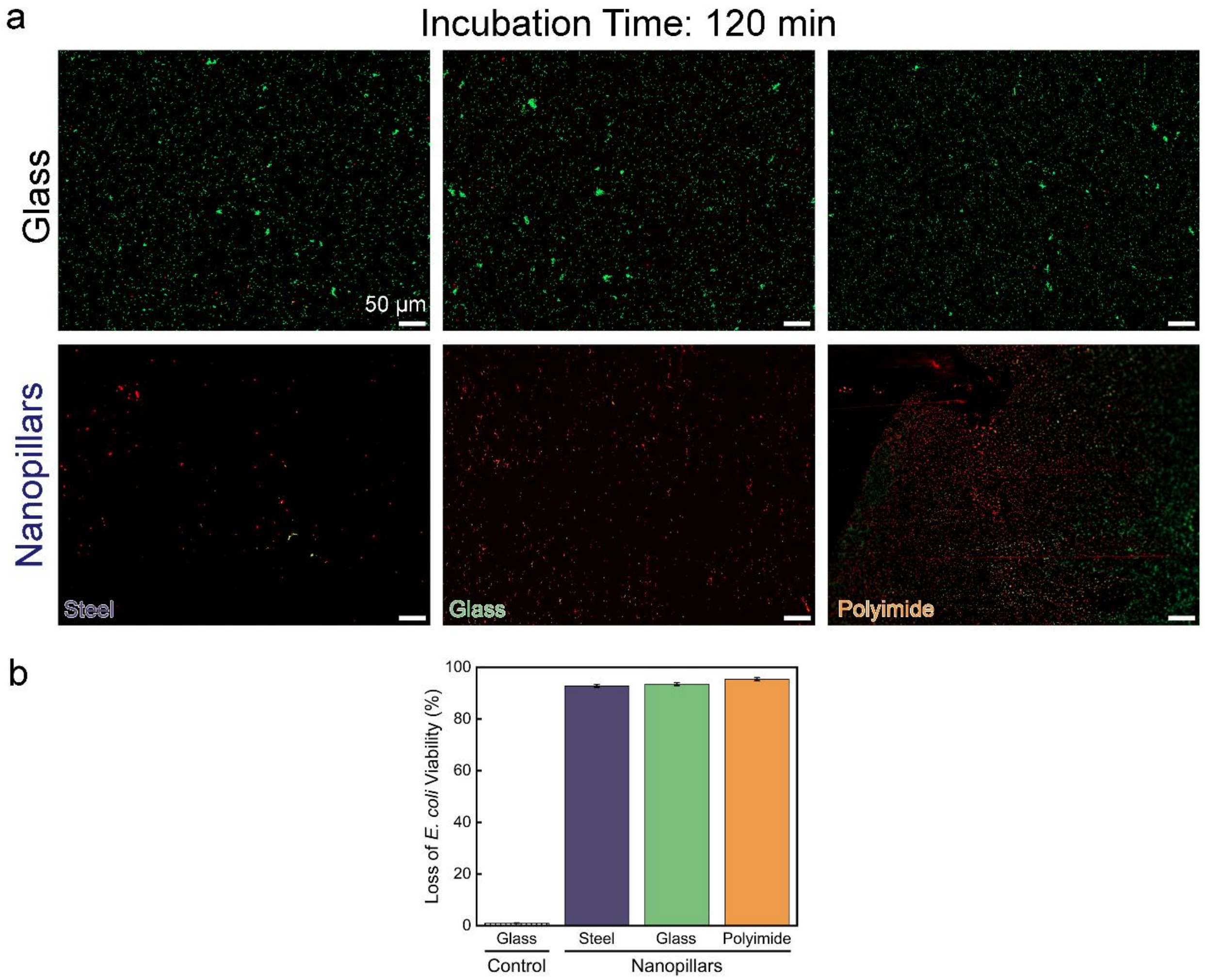

Figure S4. a Representative fluorescent micrographs of *E. coli* after a 120 min incubation on nanopillars initiated from pencil-drawn graphite that was scribbled on glass, polyimide and steel substrates. Glass controls (no nanopillars) are also displayed. Provided scale bars are 50 µm. **b** Total cell count quantified that greater than 97% of *E. coli* were inactivated after a 120 min incubation period on nanopillars initiated from pencil-drawn graphite that was scribbled on glass, polyimide and steel substrates.

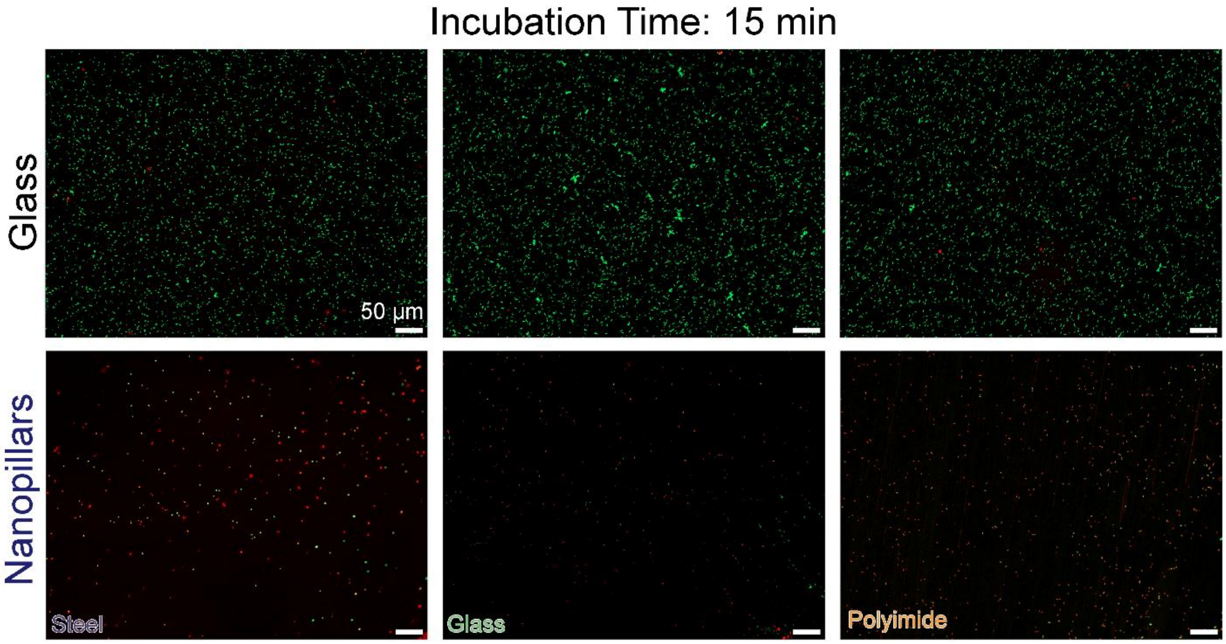

Figure S5. Representative fluorescent micrographs of *E. coli* after a 15 min incubation on nanopillars initiated from pencil-drawn graphite that was scribbled on glass, polyimide and steel substrates. Glass controls (no nanopillars) are also provided. Provided scale bars are 50 µm.
